# Classification of Soft Tissue Sarcoma Specimens with Raman Spectroscopy as Smart Sensing Technology

**DOI:** 10.34133/2021/9816913

**Published:** 2021-12-06

**Authors:** Liming Li, Vamiq M. Mustahsan, Guangyu He, Felix B. Tavernier, Gurtej Singh, Brendan F. Boyce, Fazel Khan, Imin Kao

**Affiliations:** ^1^Department of Mechanical Engineering, Stony Brook University, NY, USA; ^2^Department of Pathology and Laboratory Medicine, Stony Brook University Hospital, Stony Brook, NY, USA; ^3^Division of Plastic Surgery, Department of Surgery, Stony Brook University Hospital, Stony Brook, NY, USA; ^4^Department of Orthopaedic Surgery, Stony Brook University Hospital, Stony Brook, NY, USA

## Abstract

Intraoperative confirmation of negative resection margins is an essential component of soft tissue sarcoma surgery. Frozen section examination of samples from the resection bed after excision of sarcomas is the gold standard for intraoperative assessment of margin status. However, it takes time to complete histologic examination of these samples, and the technique does not provide real-time diagnosis in the operating room (OR), which delays completion of the operation. This paper presents a study and development of sensing technology using Raman spectroscopy that could be used for detection and classification of the tumor after resection with negative sarcoma margins in real time. We acquired Raman spectra from samples of sarcoma and surrounding benign muscle, fat, and dermis during surgery and developed (i) a quantitative method (QM) and (ii) a machine learning method (MLM) to assess the spectral patterns and determine if they could accurately identify these tissue types when compared to findings in adjacent H&E-stained frozen sections. High classification accuracy (>85%) was achieved with both methods, indicating that these four types of tissue can be identified using the analytical methodology. A hand-held Raman probe could be employed to further develop the methodology to obtain spectra in the OR to provide real-time in vivo capability for the assessment of sarcoma resection margin status.

## 1. Introduction

Soft tissue sarcomas are cancers that originate from mesenchymal progenitor cells and commonly arise in muscles and soft tissues of the extremities. The mainstay of treatment is complete surgical resection of the entire tumor bed, and the primary goal of surgery is to “leave no tumor cell behind.” Despite this goal, local recurrence (i.e., reappearance of a tumor mass despite initial surgical resection) remains a major problem in soft tissue sarcomas if they are not completely removed with negative resection margins [1].

Local recurrence occurs when tumor cells are (inadvertently) left behind during surgery. To help reduce local recurrence, it is beneficial to have a system that can quickly and reliably assess the walls of the cavity left in the surgical site after a sarcoma has been resected. Intraoperative frozen section histologic analysis is the conventional means of assessing margin status in soft tissue sarcoma surgery. Despite its widespread use, this technique is time- and labor-intensive, prone to sampling errors, and only assesses a tiny fraction of the residual surgical bed. The surgeon takes small samples of tissue from parts of the surgical cavity where the distance between the tumor and the margin is closest and sends these for frozen section histologic examination by a pathologist. It can take up to 30-45 minutes or longer for these histologic assessments to be completed, depending on the number of samples submitted and the availability of the pathologist (all while the patient is under anesthesia with an open wound). Thus, there is an unmet clinical need to develop methods that can more quickly and accurately determine if surgical margins of sarcomas are negative.

Raman spectroscopy is a noninvasive technique that can be used to obtain structural fingerprints of molecules in tissues by inelastic scattering of photons [2]. It has high chemical specificity and can obtain molecular characteristics of fresh tissue without staining or labeling [3]. The unique fingerprint of molecules in Raman spectra is able to differentiate/identify various types of tumor from normal tissue [4–7]. With the scanning-based instrumentation, Raman spectroscopy can generate a Raman map from the sample's surface for imaging [8]. These advantages attracted the attention of researchers and physicians to apply this technology to distinguish a tumor from normal tissues, such as circulating tumor cells [9] and brain [10, 11], breast [12], bladder [13], and kidney cancers [14]. However, Raman spectroscopy still has its limitations, including the time to generate a Raman spectroscopic image, 5-20 hr/mm^2^ with a resolution of 20-50 *μ*m [15, 16]. Current improvements in sampling methods have reduced the time to 30 min/mm^2^ with the Kriging interpolant and cubic spline interpolant [15]. Integration with autofluorescence can reduce the time to 10 min/cm^2^ [16]. Raman spectroscopes with a larger scanning spot size may facilitate acquisition of more comprehensive fingerprints of molecules from the sample and thus reduce scanning times. Current reports of the use of fiber optic hand-held Raman probes in various tissues in vivo [10, 17] show potential for their application for tissue assessment in the OR. Diagnostic potential in intraoperative assessment of margins of glioblastoma multiforme, the most aggressive and lethal brain cancer, has been reported [10]. Nguyen et al. [18] reported acquisition of Raman spectra from a tissue area of 0.79 mm^2^ to attempt to differentiate soft tissue sarcoma from normal fat using a hand-held probe. These findings provide support for Raman spectroscopy to be developed as a real-time in vivo tumor detection tool in surgical resection of tumors.

The main goal of this study is to develop a sensing technology using Raman spectroscopy with the ability to intraoperatively differentiate benign soft tissue from sarcoma and to give rapid and accurate feedback to surgeons to help to achieve negative margins during surgery. To this end, we carried out a pilot study to determine if Raman spectroscopy could be used to distinguish soft tissue sarcoma from adjacent benign soft tissues. The protocol includes sample preparation, spectrum acquisition, and spectral analysis/classification and is validated with the four types of tissue: tumor, muscle, dermis, and fat from human subjects with soft tissue sarcomas.

## 2. Materials and Methods

Recent studies show that peaks of Raman spectra at different wavenumbers in the fingerprint region (400 cm^−1^-1800 cm^−1^) have features that represent various vibration modes between chemical bonds in the organic molecules. These peaks give a unique signature for similar organic structures. Based on earlier research done in brain and breast cancer cases, we hypothesize that every type of soft tissue will have a distinctive unique Raman signature. These features or peaks are then used to develop methodology to classify fat, dermis, muscle, and tumor tissue owing to their different molecular structures. In this section, we explain the protocol to obtain spectra from the tissue samples and the methodology to classify the spectra.

### 2.1. Raman Spectrum Extraction

The Raman spectrum extraction procedure is divided into two stages: (1) tissue sample preparation and (2) spectrum acquisition which are described below.

#### 2.1.1. Sample Preparation

**Figure 1:**
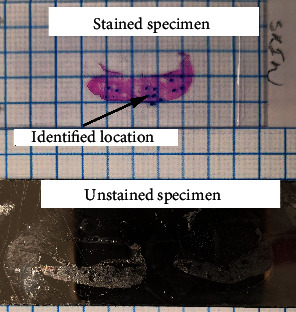
A H&E-stained tissue specimen and an unstained specimen on a stainless-steel plate.

A wide excision of a soft tissue sarcoma, which includes tumor and surrounding tissues, is performed by surgeons. Pathologists identify tumor and surrounding soft tissues and collect small pieces of the tumor, muscle, dermis, and fat from the excised specimen for frozen section and Raman analysis. Small samples from each of the four tissue types are frozen, and 10 *μ*m thick sections are cut and placed on stainless-steel plates for analysis using Raman spectroscopy. Additional sections from the same tissue blocks are cut for Hematoxylin and Eosin (H&E) staining and histologic assessment, as shown in Figure 1. Thereafter, pathologists use these sections to identify areas on the stainless-steel slides for Raman spectral microscopy.

A total of 11 samples of fresh tissues from wide excisions of three different sarcomas were procured, as illustrated in Table 1. We cut sections of these tissues for H&E staining and Raman spectroscopy as explained above. Based on the H&E staining, the pathologist identified forty-seven areas in these slides, including tumor, muscle, dermis, and fat tissues. The distribution of these areas for the three specimens is tabulated in Table 2.

#### 2.1.2. Spectrum Acquisition

**Figure 2:**
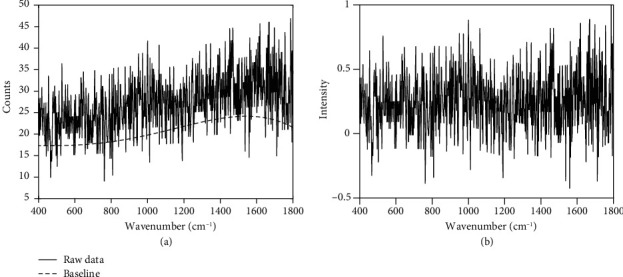
(a) Raw data of the Raman spectrum and (b) adjusted data of the Raman spectrum after baseline correction and normalization of intensity.

A Renishaw inVia Confocal Raman Microscope is used to acquire the Raman spectra from a spot within the identified locations in the unstained samples. Each exposure at a spot can only illuminate an area with a diameter less than 1 *μ*m, which is much smaller than the size of a cell. Considering that different types of tissue have various compositions, a 100 *μ*m × 100 *μ*m area is scanned to obtain 2601 Raman spectra (51 × 51 spots) to obtain comprehensive analysis of the tissue for each measurement. Each spectrum is obtained through an exposure of 0.5 second at each spot by the 633 nm He/Ne laser (17 mW). The baseline in each spectrum is removed by the fourth order of polynomial and normalized to 1, as shown in Figure 2. The spectrum we refer to in this paper has a horizontal axis of wavenumbers in a unit of cm^−1^, ranging from a few hundreds to about 1800, and a vertical axis that represents the intensity of the signal at each wavenumber, as illustrated in Figure 2. The intensity is adjusted to zero using a baseline curve and normalized with the maximum signal to 1.0. It is noted that the spectrum is digitized with discrete wavenumbers in the horizontal axis with corresponding intensity in the vertical axis. Table 2 shows the source and distribution of a total of 120,184 spectra in the study.

## 3. Classification Methods for Diagnosis and Results

Two different and independent methods for diagnosis and tissue classification are presented to process the spectra in order to classify the types of tissue. The two methods are (1) a quantitative method and (2) a machine learning method and are presented in the following sections.

### 3.1. Classification Using the Quantitative Method

The first method is the quantitative method to classify the Raman spectra with the associated type of tissue through comparison with reference spectra. Four types of reference spectra are established for the tumor, muscle, dermis, and fat, respectively.

#### 3.1.1. Generate the Reference Spectrum

**Figure 3:**
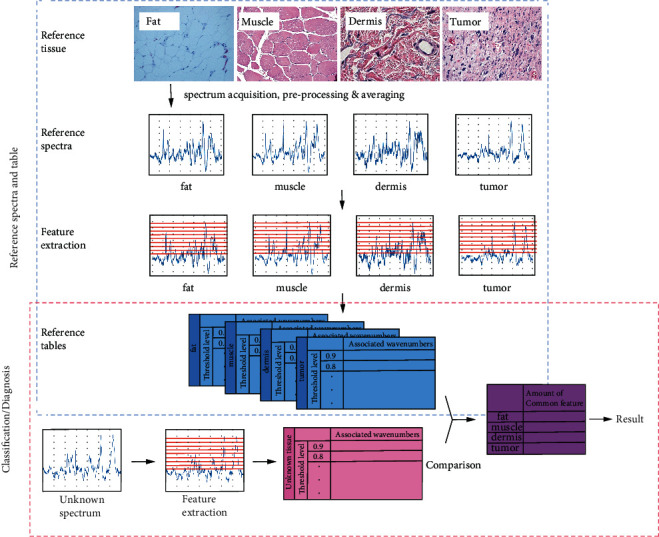
Flow chart of the quantitative method. The first group of the top four rows constitutes the reference and feature extraction, as described in Sections 3.1.1 and 3.1.2. The second group of the last two rows is for the classification using the reference tables, as described in Section 3.1.3.

All spectra used to generate the reference spectrum have a corrected baseline and normalized intensity by the preprocessing described in Section 2.1.2. The processed spectra from the same tissue are averaged to establish the reference spectrum. This process is repeated for all different types of tissue to generate the four reference spectra for the tumor, muscle, dermis, and fat tissues. The steps are illustrated in the first two rows in Figure 3.

#### 3.1.2. Extract Features of the Reference Spectrum

**Figure 4:**
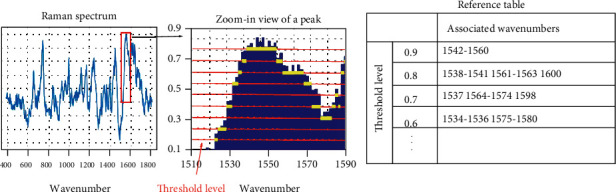
Illustration of feature extraction of the quantitative method showing the following: (left) isolating a peak from the reference spectrum, (middle) identifying the wavenumbers associated with each threshold level (in green color), and (right) generating the reference table of threshold levels and their associated wavenumbers.

In order to extract the features from the reference spectrum, several threshold levels are defined to divide the normalized intensity into several intervals at a range of wavenumbers corresponding to a peak. In this study, nine threshold levels from 0.1 to 0.9 are used, with an interval of 0.1 and a total of 10 intervals. This is illustrated in Figure 4 with the corresponding threshold levels from 0.1 to 0.9. The example in Figure 4 shows a peak isolated from the reference spectrum with a range of wavenumbers from 1510 to 1590 cm^−1^. Next, if an intensity level falls between two threshold levels, the corresponding wavenumbers are recorded as associated with the lower threshold level. For example, the intensity in the range of wavenumbers of 1542-1560 cm^−1^ is between 0.9 and 1.0; hence, the wavenumbers of 1542-1560 cm^−1^ are recorded as associated with the threshold level of 0.9. A reference table of the wavenumbers associated with each threshold level is constructed to represent the features of the reference spectrum for each type of tissue as the database for comparison. This is shown in the “reference table” in Figure 4 with the threshold levels and associated wavenumbers. Figure 3 shows the steps to establish the reference database table by extracting features of the reference spectrum. The reference tables for all four types of tissues are generated.

#### 3.1.3. Classification of an Unknown Spectrum

An unknown spectrum of a tissue type is first processed to have a baseline correction and normalization using the same procedures in Section 3.1.1. With the same threshold levels in feature extraction as described in Section 3.1.2, the features of the unknown spectrum can be extracted to form a table of the threshold levels and the associated wavenumbers. This table of values of unknown tissue is compared with one reference table of the reference spectrum of one tissue type obtained in Section 3.1.2. The total numbers of matched wavenumbers associated with each threshold level in both tables are counted and tallied for all threshold levels. This comparison is repeated for the remaining three reference tables of tissue types. The total counts of matches of the unknown spectra with respect to each of the four reference tables of all tissue types are compared. The highest counts of matches, among the comparison of four reference tables, determine the match with the corresponding tissue type for the classification. Results of classification will be presented later in Section 4.3. The procedures of classification are shown in the last two steps of the flow chart in Figure 3.

#### 3.1.4. Experiments to Classify Unknown Spectra

In the experiment with the data of the three patient samples, we used 80% of the spectra for each tissue type to generate the reference spectrum by averaging and establishing the reference tables. This process gives us eleven reference tables, one for each of the four tissue types from each patient. The remaining 20% of spectra are used as unknown spectra in a blind test of classification using the procedures in Section 3.1.3. Therefore, we have a total of 46 average unknown spectra for the experiment and 11 reference tables for the reference database.

### 3.2. Classification Using Machine Learning

In addition to the quantitative method described in Section 3.1, we used machine learning independently to classify the signature of Raman spectra from a particular tissue. Using multiple techniques in tandem helps increase the confidence in the classification result and improves robustness of classification. Two types of machine learning networks are presented in the following sections.

#### 3.2.1. One-Dimensioned (1D) Machine Learning Network

For the purpose of developing a 1D machine learning network, we consider only the intensity values without the associated wavenumber values. The initial database is created with tissue labels, based on the locations in the sections on the metal slides identified by the pathologist. Each wavenumber is considered an independent variable in the 1D machine learning model. The corresponding intensity values are preprocessed before being input into an Artificial Neural Network (ANN) classifier [19–25].

**Figure 5:**
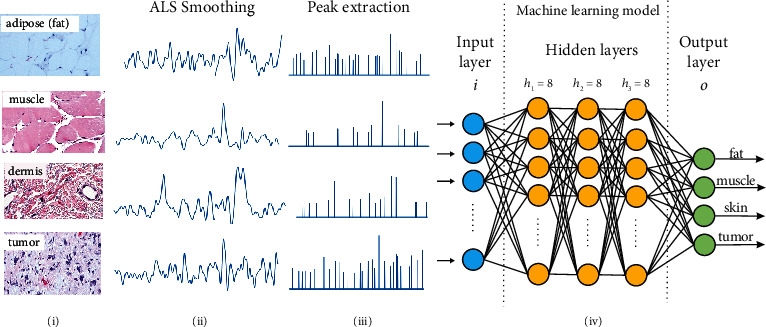
Schematic illustration of the 1D machine learning network for classification: (i) H&E illustration of adipose, muscle, dermis, and tumor tissue; (ii) Raman spectra obtained from the respective tissue type after baseline subtraction and ALS smoothing; (iii) representation of the feature vector obtained after peak extraction from the smoothed Raman spectra which are eventually input into the ANN model; and (iv) ANN architecture used to classify the input spectrum.

In the preprocessing steps, we took the input data obtained using a 4th-order polynomial baseline subtraction in Section 2.1.2 and performed the “asymmetric least square” (ALS) smoothing to obtain a smooth spectrum and remove noise. The resultant spectrum is then passed through a threshold filter where the normalized intensities below 0.3 are removed. This process removed any valleys in the spectrum because it had been established that the peak values in the spectra are the distinct features for molecular compounds. Thereafter, these peaks in the resultant signals are extracted and saved as a feature vector for every spectrum. The resultant vectors were used as input to train the ANN classifier. The 1D ANN classifier has an input layer which takes in the intensity vector and is followed by 3 hidden layers with 8 nodes of which each leads to the output layer [19, 20, 22–25]. This is illustrated in Figure 5.

#### 3.2.2. Two-Dimensional (2D) Machine Learning Network

The 1D network in Section 3.2.1 only considers the vector of intensity values as input without the wavenumbers. In the design of a 2D network, we include both the wavenumbers and the corresponding intensity values as 2D input data to the network. This 2D input is sent through the same preprocessing steps explained in Section 3.2.1. After the peaks are extracted, we resize the input to a 650 × 2 array to fit into the Convolution Neural Network (CNN) architecture [26, 27].

**Figure 6:**
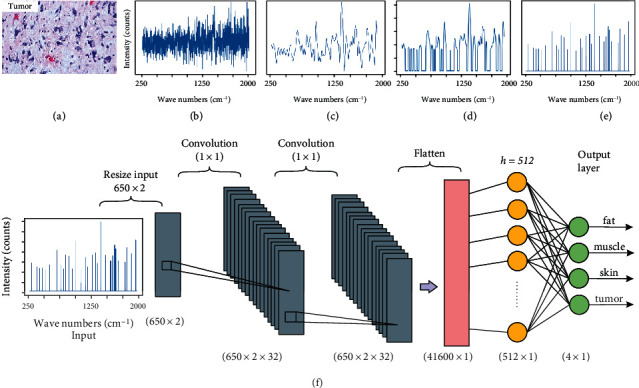
(a) H&E-stained section of high-grade sarcoma, (b) spectrum obtained after baseline correction, (c) spectrum after performing ALS smoothing, (d) removing valleys in the spectrum by applying a threshold filter, (e) extraction of peaks at their corresponding wavenumbers, and (f) CNN model where the peak intensity values and wavenumbers are introduced to the 2D CNN architecture.

The resultant spectrum input into the 2D CNN classifier has the peak intensity values along with the corresponding wavenumbers. This input is passed through a network of 32 (1 × 1) convolution filters twice, as shown in Figure 6(f), and is eventually flattened and passed through one hidden layer with 512 nodes. The hidden layer is followed by an output layer which gives the classification of the spectrum. The 2D CNN classifier model is shown in Figure 6.

#### 3.2.3. Experiments to Classify Unknown Spectra

The 1D ANN classifier was trained using 16,400 spectra and validated using 7000 spectra, all from samples from patient #1. However, when we obtained spectra from the tissue from patients #2 and #3, we realized that the range of wavenumbers of the spectra recorded was different from that from patient #1. Therefore, including spectra from different patients in the 1D ANN (explained in Section 3.2.1) would not result in a consistent database without including the information of the wavenumbers in the spectra.

In light of this, we developed the 2D CNN classifier which was able to preserve the relationship between wavenumbers and intensity values and successfully compiled the data of all 3 patients. The training is done with 70% of the spectra (84,184) and tested on the remaining 30% of the spectra.

## 4. Discussion

### 4.1. Consistency in Various Types of Tissue

**Figure 7:**
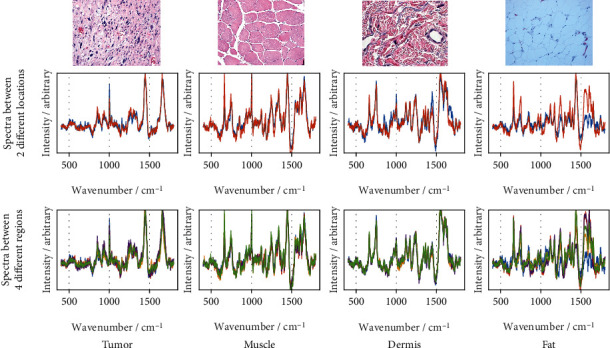
Consistency of spectra: (a, top) H&E-stained sections of the tumor, muscle, dermis, and fat; (b, middle) plots of the average of Raman spectra at two different locations of the same tissue type; and (c, bottom) plots of the average of spectra at two different locations, each having 4 regions.

In order to establish the reference database for the quantitative method and to have a stable model in the training of machine learning networks, the Raman spectra obtained from a specific type tissue should demonstrate a consistent pattern for classification. Such consistency should be observed at different scanning locations for the same tissue type. To this end, we scanned two different locations for each of the four types of tissues from patient #1 to observe if there is consistency in the spectra from the two different locations. The results of comparison are plotted in Figure 7(b), with two spectra, which show very good similarity of spectra from different locations in the tissues, especially for the tumor and muscle.

In addition, we divided each location (each location has an area of 100 *μ*m × 100 *μ*m. The location is divided into four equal regions, each with an area of 50 *μ*m × 50 *μ*m. A location has a total of 2601 spectra, and a region within a location has a total of 650 spectra) into 4 regions and obtained the average of the spectra of each region. We compared the spectra at different locations and different regions, as shown in Figure 7(c), with 8 different spectra.

The spectra from the tumor and muscle have good consistency in both peaks and characteristics, as shown in Figures 7(b) and 7(c). Although they share most of the peaks, the spectra of the dermis and fat each show some different peaks, such as in the region of 1650-1700 cm^−1^ for the dermal spectra and the peak 1520-1640 cm^−1^ for the fat spectra.

In summary, the spectra from different areas of the same tissue show consistency. The distinct features of different types of tissues are also consistent to provide reliable classification.

### 4.2. Features of Spectra in Different Types of Tissue

**Figure 8:**
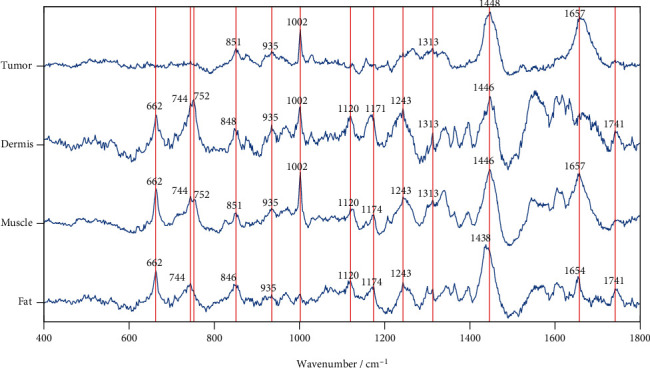
Comparison of peaks among the spectra of the tumor, dermis, muscle, and fat.

Figure 8 shows typical spectra of the tumor, dermis, muscle, and fat, which share peaks at the following wavenumbers: 846-851, 935, 1002, 1313, 1438-1448, 1657, and 1741 cm^−1^. However, the intensities at the common peaks are different. For example, the peak at 1002 is smaller for fat. The tumor and muscle have a small peak at 1741 cm^−1^. In addition, different types of tissue can have distinct peaks in their spectra. We can clearly observe that there is no peak at 662, 744, and 752 cm^−1^ in tumor tissue. Dermis and fat have three peaks at 1343, 1364, and 1396 cm^−1^, while muscle has a higher peak at 1343 cm^−1^ than the peaks at 1364 and 1396 cm^−1^. However, the tumor does not show peaks at 1364 and 1396 cm^−1^. Although they share some common peaks, different spectra in all types of tissue have their own distinct features for classification.

### 4.3. Results of Classification

As described in Section 3.1.4, the unknown spectra in a blind test are compared against the reference spectra of the tumor, muscle, dermis, and fat using the quantitative method with the reference tables which contain the threshold values and the associated wavenumbers. The results of classification using the quantitative method are in Table 3. The results of blind tests demonstrated that the quantitative method was able to correlate the unknown spectra with the reference correctly with an overall accuracy *P*_q_ = 83.0% (39/47) for the classification of all patients. For example, the unknown sample in the first column of Table 3 (which is from tumor tissue) results in 14 counts of match with the tumor and with the fat. This result gives a *P*_q_ = 93.3% (14/15) accuracy that the unknown sample is tumor tissue. The blind spectra in the second column of Table 3 have 12 counts of match with the muscle with 2 counts of match with the fat and 1 count with the tumor. This suggests that the unknown sample has a *P*_q_ = 75% (9/12) likelihood to be muscle.

The results of classification using the 1D ANN classifier on test data obtained from patient #1's sample (7000 spectra) have an accuracy of 73.7%, as illustrated in Table 4. However, this accuracy increased to 85.8% when we used an averaged spectrum over the area. Furthermore, when this model was applied to patient #2 and patient #3's samples, we observed low accuracy of about 34%. When the 2D machine learning method was applied, as explained in Section 3.2.2, the training of the classifier includes 2D information of both the intensity and the corresponding wavenumbers. The accuracy of classification improves to *P*_m_ = 85.8% (percentage of correct classification from the test spectra) with sensitivity of 94.9% (true positive rate) and specificity 88.3% (true negative rate) for tumor detection. The results are tabulated in Table 5. Sensitivity of the classifier can be defined as the percentage of positives that are correctly identified.

(1) $$\begin{array}{c} \text{Sensitivity}=\frac{\text{True positives detected}}{\text{Actual number of positives}}. \end{array}$$

Specificity of the classifier can be defined as the percentage of negatives that are correctly identified.

(2) $$\begin{array}{c} \text{Specificity}=\frac{\text{True negatives detected}}{\text{Actual number of negatives}}. \end{array}$$

The results of the 1D classifier demonstrate that the averaged spectra over a larger area give more fidelity in the classification of results. On the other hand, the 2D CNN classifier, including the 2D information, preserves the relationship between the wavenumbers and the intensities of the Raman spectra and yields better results of classification.

#### 4.3.1. Comparison with Existing Results

Raman spectroscopy has been employed in different applications for the identification of tumors occurring in the brain, soft tissue, and bladder and in round blue cell tumors, with different levels of reported accuracy, depending on the tissue types and techniques used [10, 18, 28–31]. A comparison of current research on the tumor using Raman spectroscopy can be found in Table 6, with publications listed in reverse chronological order. Types of tumors and their accuracy, sensitivity, and specificity of classification are listed, based on the available data from the publications.

As a comparison, the quantitative method presented in this paper has an accuracy of 83.0%, with a sensitivity and specificity of 93.3% and 90.6%, respectively. The machine learning method presented in this paper has an accuracy of 85.8% and sensitivity and specificity of 94.9% and 88.3%. While the two methods are individually comparable to existing methods in accuracy of classification, when both methods are used for classification collaboratively, as will be presented in Section 4.4, the accuracy and confidence of classification can be much improved over the accuracy of existing methods.

### 4.4. Using Multiple Methods for Classification

To enhance the confidence of the diagnostic outcome, the two methods proposed herein, namely, the quantitative and machine learning methods, can be employed in tandem to provide robustness and redundancy in diagnosis and classification of tumor tissue. Equation (3) is defined to calculate the likelihood of a spectrum being cancerous by combining the individual accuracy of the two methods. Weights are also defined to quantify the contribution of individual accuracy from both methods. The overall likelihood of an unknown spectrum being from a tumor tissue, *P*, is defined as follows:

(3) $$\begin{array}{c} P=1-\left[\frac{2W_{\text{q}}}{W_{\text{q}}+W_{\text{m}}}\left(1-P_{\text{q}}\right)\times \frac{2W_{\text{m}}}{W_{\text{q}}+W_{\text{m}}}\left(1-P_{\text{m}}\right)\right], \end{array}$$

where *P*_q_ and *P*_m_ are the accuracy obtained from the quantitative and machine learning methods, respectively, and *W*_q_ and *W*_m_ are the weights of contribution of the quantitative and machine learning methods, respectively, typically adding up to 100%; that is,

(4) $$\begin{array}{c} W_{\text{q}}+W_{\text{m}}=1.0. \end{array}$$

The accuracy is calculated using a straightforward ratio in Sections 4.3. For example, the accuracies are *P*_q_ = 83.0% and *P*_m_ = 85.8% from Section 4.3 for the quantitative and machine learning methods, respectively. If an equal weight of *W*_q_ = *W*_m_ = 50% is chosen, we can calculate from equation (3):

(5) $$\begin{array}{c} P=1-\left[2\times 0.5\left(1-0.830\right)\times 2\times 0.5\left(1-0.858\right)\right]=0.976=97.6\%. \end{array}$$

With the redundant diagnosis using two independent methods, a confidence of 99.7% is achieved for the identification of the tumor. Equation (3) can be extended easily to multiple classifiers to determine the confidence of a classification.

### 4.5. Feasibility of Using a Hand-Held Probe

As explained in the earlier section, the Renishaw spectroscope is a confocal spectroscope which takes about 30 min to scan a 100 *μ*m × 100 *μ*m area with the exposure of 0.5 sec for 2601 spectra. The spectra are averaged to obtain reference data for classification. The current hand-held optical fiber probe can illuminate an area with a diameter greater than 200 *μ*m in one measurement, which is much larger than the scanning area in our study. It is postulated that a probe with larger spot size can obtain spectra in a shorter time that has an equivalent effect of averaging many of the spectra in this study. We hypothesize that the average spectrum of a larger area will be able to better represent the tissue and increase the accuracy of classification.

To determine the feasibility of using this probe, we divided the scanned areas of patient #1's sample into smaller regions of 2, 4, 9, 16, and 25 divisions, where each division includes 1300, 650, 290, 150, and 110 spectra, respectively. In each case, we calculate the accuracy of classification to observe the consistency in detection. In this planned study, we will also be able to determine how much area needs to be scanned to accurately differentiate among 4 average spectra. This will prove the feasibility of using the hand-held probe which measures the Raman spectrum of a larger area.

**Figure 9:**
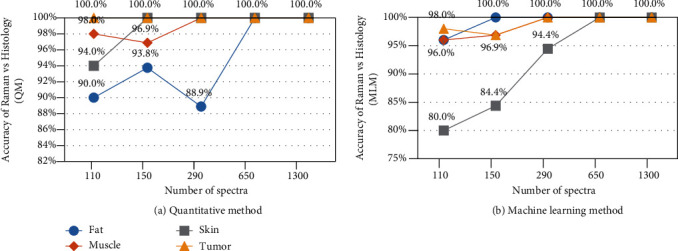
Accuracy of classification versus the number of spectra using (a) the quantitative method (QM) and (b) the machine learning method.

**Figure 10:**
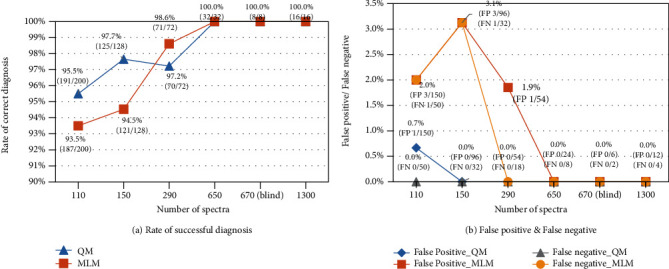
(a) Rate of correct classification and (b) false positives and false negatives with different numbers of spectra using the quantitative method (QM) and the machine learning method (MLM).

The results in Figure 9 show that the rate of correct classification increases with the increase in the number of spectra in the scanned area. When the scanned area was divided into 4 regions (each with 650 spectra), both the quantitative and 1D ANN machine learning methods were able to successfully classify all spectra.  Figure 10(a) plots the rate of successful classification using both methods, showing the same trend of increase in accuracy of classification as the number of spectra increases. In addition, the false negative and false positive trends of both classification methods have been improved, as shown in Figure 10(b).

## 5. Conclusion

The objective of this study is to develop a means of sensor technology which can classify tumor and adjacent benign soft tissues using the Raman spectrum of these tissues. The results suggest that this sensing technology can have a similar capability as frozen section analysis by pathologists to identify tumor and adjacent soft tissues and could be further developed into a real-time and in situ tumor detection tool. This could help surgeons to be certain that they have completely removed cancer tissue using a hand-held Raman spectroscope in the operating room (OR), providing that we can demonstrate that it has a shorter time lag to classification of tissues as conventional pathological examination using small portions of tissue.

Two independent methods for classification of tumor and adjacent soft tissues are presented: the quantitative method and the machine learning method. Both methods demonstrate high accuracy of classification. Furthermore, more accurate classification can be achieved by combining the results of the two accuracy measures from both methods to enhance the confidence of classification.

The spectra are shown to be consistent within each tissue type. Individual tissue types, nevertheless, have their own distinct features and characteristic peaks. The consistency within the spectra of each tissue type and certain distinct features that differentiate different tissue types make the classification of tissue more robust with consistent outcomes.

Finally, we presented the preliminary results of a study to determine the feasibility of using a hand-held probe by examining the rate of successful classification. The results are promising for future study.

## Figures and Tables

**Table 1:** The number of fresh tissue samples of patients obtained for the study.

	Tumor	Muscle	Dermis	Fat
Patient #1	1	1	1	1
Patient #2	1	1	0	1
Patient #3	1	1	1	1
Total	3	3	2	3

**Table 2:** The number of scanned areas in each type of tissue and their corresponding spectra extracted from the patients.

	Tumor	Muscle	Dermis	Fat	No. of spectra
Patient #1	2	2	3	2	23,409
Patient #2	4	4	0	4	30,995
Patient #3	9	6	7	4	65,780
Total scanned areas	15	12	10	10	
Total spectra	39,142	29,055	25,989	25,998	120,184

**Table 3:** Results of classification using the quantitative method.

	Blind spectra (tumor)	Blind spectra (muscle)	Blind spectra (dermis)	Blind spectra (fat)
Tumor reference	14	1	2	0
Muscle reference	0	9	0	0
Dermis reference	0	0	7	1
Fat reference	1	2	1	9

**Table 4:** Results of classification using the machine learning, with blind test spectra from patient 1 using the 1D ANN classifier.

	Blind spectra (fat)	Blind spectra (muscle)	Blind spectra (dermis)	Blind spectra (tumor)
Fat, trained model	1108	98	342	4
Muscle, trained model	40	1126	209	176
Dermis, trained model	177	230	1722	195
Tumor, trained model	17	68	270	1200

**Table 5:** Results of classification using the machine learning, with blind test spectra from patients using the 2D CNN classifier.

	Blind spectra (fat)	Blind spectra (muscle)	Blind spectra (dermis)	Blind spectra (tumor)
Fat, trained model	7011	110	250	367
Muscle, trained model	291	7735	51	562
Dermis, trained model	508	52	5395	1898
Tumor, trained model	194	133	269	11,230

**Table 6:** Summary of accuracy, sensitivity, and specificity for the identification of the tumor using Raman spectroscopy for comparison.

Authors & year	Type	Accuracy (%)	Sensitivity (%)	Specificity (%)
Hollon et al. 2020 [28]	Brain tumor	94.6		
Jeng et al. 2019 [29]	Brain tumor	81.25-87.5	77.27-90.90	86.11-83.33
Nguyen et al. 2016 [18]	Soft tissue sarcoma		89.5	96.4
Jermyn et al. 2015 [10]	Brain tumor		93	91
Kast et al. 2010 [30]	Round blue cell tumor	87.9-100		
de Jong et al. 2006 [31]	Bladder tumor	93	94	92

## Data Availability

Data of this paper are available by emailing imin.kao@stonybrook.edu.
